# Adenoid Ameloblastoma: A Case Report on this Newly Recognized Under-Treated Odontogenic Tumour

**DOI:** 10.1007/s12663-025-02592-3

**Published:** 2025-07-03

**Authors:** Fadi Titinchi, Moegamat Sallies, Johan Opperman

**Affiliations:** 1 Department of Maxillo-Facial and Oral Surgery, Faculty of Dentistry and WHO Collaborating Centre, University of the Western Cape, Private Bag X1, Tygerberg Oral Health Centre, Francie Van Zijl Drive, Cape Town, 7505 South Africa; 2 Department of Oral and Maxillofacial Pathology, University of the Western Cape and National Health Laboratory Service, Cape Town, South Africa; 3 Department of Diagnostic and Surgical Dental Sciences, College of Dentistry, Gulf Medical University, Ajman, United Arab Emirates

**Keywords:** Ameloblastoma, Adenomatoid odontogenic tumour, Resection, Fibula free flap

## Abstract

**Introduction:**

Adenoid ameloblastoma (AdAM) is a rare epithelial odontogenic tumour recently included in the 5th World Health Organization classification of head and neck tumours as a separate tumour from conventional ameloblastoma. It presents diagnostic and management challenges due to its morphological overlapping features with adenomatoid odontogenic tumour (AOT) and conventional ameloblastoma.

**Case Report:**

A 32-year-old female with neurofibromatosis type I presented with a 4-month history of right-sided facial swelling. Clinical examination revealed a firm, expansive mass in the right anterior mandibular region with associated tooth mobility. Radiographic evaluation demonstrated a well-demarcated unilocular radiolucency mimicking a cystic lesion. Histological examination revealed an epithelial odontogenic neoplasm composted of nests of ovoid to spindle-shaped cells demonstrating pseudo-duct-like spaces, cribriform areas and a whirling pattern with deposition of dentinoid material. These features confirmed the diagnosis of AdAM. The tumour was resected and immediately reconstructed with a fibula free flap. The patient remains diseases-free 2 years post-surgery.

**Conclusion:**

There are some overlapping clinico-pathological features of this tumour with AOT, which has led to inadequate surgical management previously with high recurrence rates. Emphasis should be placed on identifying all histopathological features described to arrive at an accurate diagnosis. Surgical treatment of AdAM should be radical resection despite its clinical and radiographic presentation mimicking the less aggressive AOT.

## Introduction

In 2022, the World Health Organization (WHO) updated its classification of head and neck tumours to include adenoid ameloblastoma (AdAM) as a benign epithelial odontogenic tumour distinct from conventional ameloblastoma. This lesion is exceptionally rare with approximately 40 reported cases in the literature and is histologically characterised by the presence of ameloblastomatous epithelium, cribriform architecture, duct-like structures and epithelial whorls (morules), in addition to deposition of dentinoid material, ghost cells and clusters of clear cells. Due to overlapping radiographic and histological features, it has been confused with adenomatoid odontogenic tumour (AOT) and conventional ameloblastoma [1]. AdAM is a locally invasive tumour known for its aggressive clinical behaviour and high recurrence rate of approximately 70% with conservative treatments [2]. Herein, we report on a rare case of AdAM highlighting its similarities to AOT and conventional ameloblastoma as well as its definitive surgical management.

## Case Report

### Patient Information

A 32-year-old female known with neurofibromatosis type I presented with a 4-month history of a right-sided facial swelling.

### Clinical Findings

A firm expansive mass was palpable in the right anterior mandibular region, and mobility of the associated dentition was noted.

### Timeline

See Fig. 1.

**Fig. 1:**
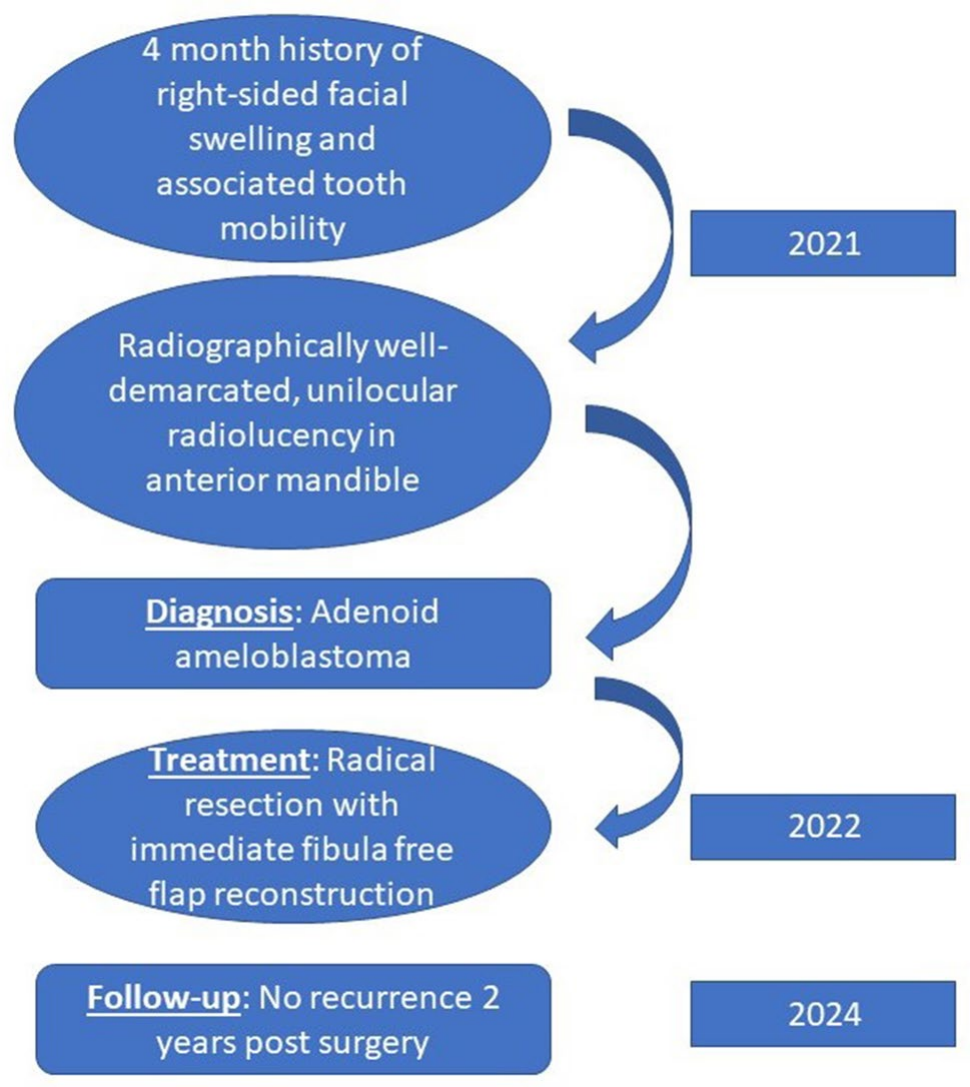
Case report timeline according to CARE guidelines

### Diagnostic Assessment

**Fig. 2:**
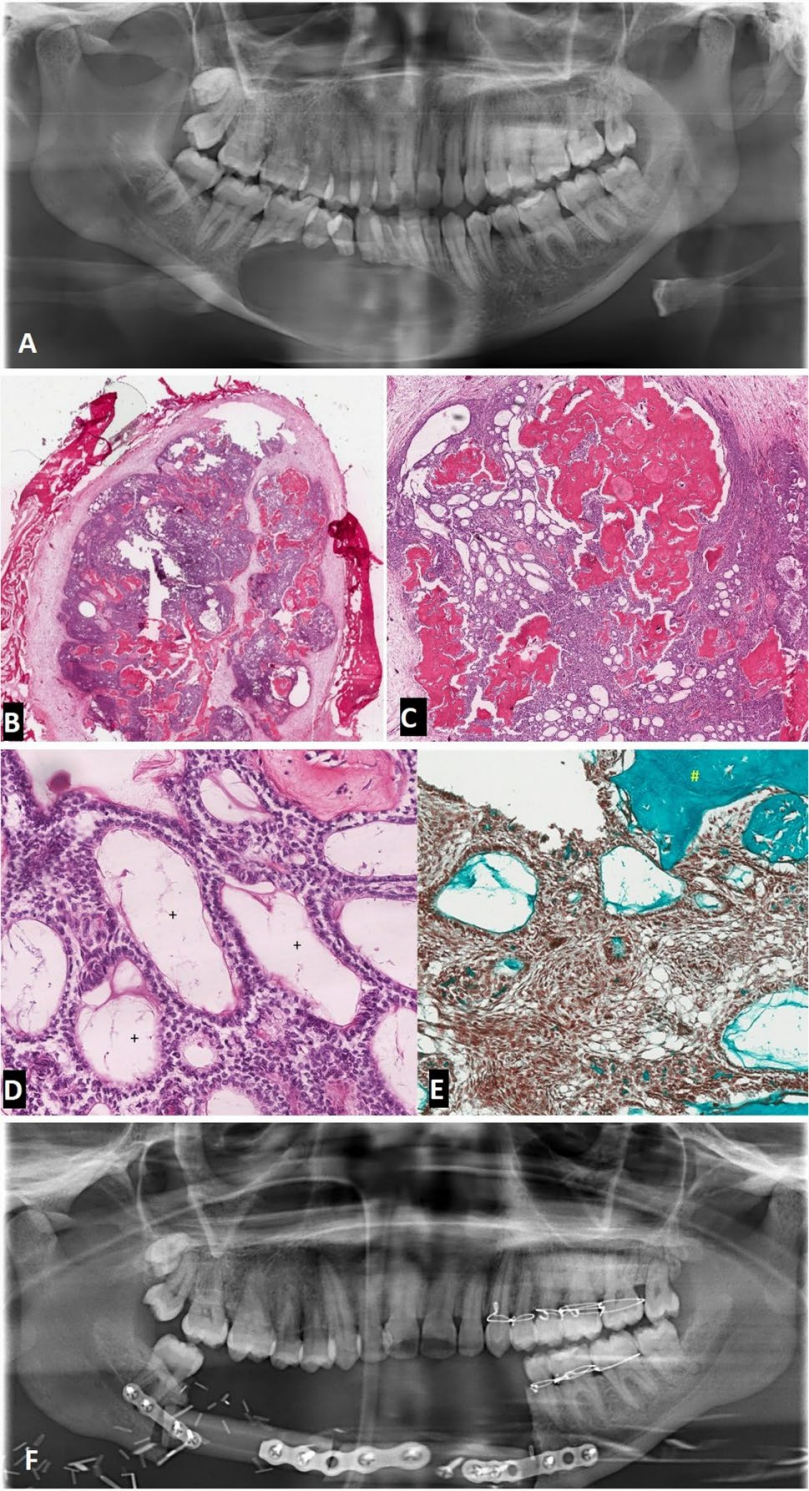
**A** Pre-operative pantomograph showing unilocular radiolucency in the right mandible mimicking cystic tumour. **B** H & E-stained specimen shows a well-circumscribed tumour surrounded by a bony rim. (20 × magnification). **C** Basaloid odontogenic tumour islands with pseudo-glandular structures and “pink” dentinoid material. (40 × magnification). **D** Tumour islands showing peripheral basal cell palisading, reverse nuclear polarity with pseudo-glandular structures (+). **E** Masson–Trichrome stain highlights the dentinoid material (turquoise #). (200 × magnification). **F** Post-operative pantomograph following radical resection and reconstruction with fibula free flap

Radiographic examination demonstrated a well-demarcated expansile lytic lesion centred in the right mandibular body measuring approximately 46.5 mm x 30 mm x 30 mm (Fig. 2A). On contrast-enhanced computed tomography, limited punctate calcified foci were easily visualized within the tumour matrix and soft tissue perforation could be seen through complete erosion of the bony cortex. Additionally, the right buccal and lingual cortices demonstrated varying degrees of thinning and demineralization.

The patient underwent an incisional biopsy and extraction of the associated dentition under general anaesthesia. Microscopic sections revealed an epithelial odontogenic neoplasm composted of nests and sheets of ovoid to spindle-shaped cells demonstrating pseudo-duct-like spaces, cribriform areas and a whirling pattern (Fig. 2B, C). The cells at the periphery were palisaded, exhibiting reverse nuclear polarity (Fig. 2D). In the centre of the nests were areas resembling stellate reticulum cells and interspersed areas of eosinophilic dentinoid material. Ghost cells and clear cells were absent. Furthermore, sections were negative for both BRAF and CD56 immunohistochemical stains but focally positive for calreticulin (CALR). Both Masson Trichrome and Van Gieson histochemical stains highlighted the dentinoid material (Fig. 2E). This confirmed the diagnosis of an adenoid ameloblastoma with dentinoid.

### Therapeutic Intervention

The patient underwent resection with a 1-cm-wide margin and immediate reconstruction with a fibula free flap (Fig. 2F).

### Follow-Up and Outcomes

At 2 years post-resection, there was no evidence of tumour recurrence as observed at follow-up visits, and the patient has since been prosthodontically rehabilitated and remains symptom-free.

## Discussion

Adenoid ameloblastoma (AdAM) is a recently recognized tumour that has raised increasing attention in the last decade. Despite recommendations for its classification as a distinct entity, it remains underdiagnosed due to its overlapping clinical, radiographic, and histopathologic features with other odontogenic tumours, particularly conventional ameloblastoma and adenomatoid odontogenic tumour (AOT). This diagnostic uncertainty is critical, as accurate identification is essential for appropriate management and prognosis. Misdiagnosis often leads to inappropriate treatment, which may influence recurrence rates and patient outcomes [1].

AdAM presents predominantly in adults (76.5%) and more frequently in males (63.3%) with mean age of 40.8 years and ages spanning from 15 to 70 years [1]. Most cases occur in the fourth decade with the age distribution pattern resembling that of conventional ameloblastoma more than AOT, which typically occurs in the first or second decades of life. Only one case was reported in a patient below 18 years of age [1].

AdAM cases are slightly more common in the mandible (53.3%) than in the maxilla (46.7%), a characteristic shared with conventional ameloblastoma. Furthermore, most AdAMs involve the posterior regions of the mandible. These findings suggest that while AdAM shares some features with AOT, its age and anatomical distribution patterns align more closely with the behaviour of conventional ameloblastoma, which are more aggressive in nature [3].

The majority of reported cases present as asymptomatic swelling, with only few cases reporting accompanying pain. A minority of cases exhibit paraesthesia, all involving the mandibular posterior region. While pain and paraesthesia are less common in conventional ameloblastoma, they may occur due to tumour mass affecting peripheral nerves or due to secondary infection. The presence of symptoms in AdAM further underscores its potentially aggressive behaviour, which distinguishes it from the more indolent nature of AOT [3].

Radiographically, AdAM typically appears as a well-defined unilocular radiolucency, a characteristic shared with AOT [3]. However, deviations from this typical presentation have been observed. Loyola et al*.* [4] reported two cases of poorly defined radiolucent lesions in the maxilla involving the nasal and paranasal sinuses as well as the orbit. These atypical radiographic findings suggest that AdAM may exhibit more diverse radiologic characteristics, potentially complicating its diagnosis [5].

Multilocularity was infrequently observed and only noted in two out of eight cases reported by Adorno-Farias et al. [5]. Additionally, three cases exhibited radiopaque foci within the unilocular radiolucency in the mandible as seen in our patient. Histopathologic examination of these radiopaque foci revealed ghost cells and dystrophic calcifications, which are features typically associated with more aggressive tumour behaviour [5]. de Farias Morais et al. [1] reported that 20% of AdAM exhibit cortical perforation, which was also noted in our case. This further highlights the invasive nature of this lesion.

Based on the limited literature available, the primary diagnostic criteria for AdAM encompass various indicators. These include the presence of any histopathological subtype of ameloblastoma, or the identification of a tumour containing cells resembling ameloblasts and/or stellate reticulum cells, even in the absence of the characteristic architectural arrangement typical of ameloblastoma. Additionally, diagnostic considerations may involve the observation of an AOT exhibiting at least one feature such as duct-like structures, glandular differentiation, or epithelial whorls, coupled with evidence of local invasion. These features are usually accompanied by local invasion, a key distinguishing feature of AdAM. The presence of dentinoid within a mature fibrous stroma is another significant finding [1].

The molecular basis and pathogenesis of AdAM are not well understood. While there is morphological overlap with AOT and conventional ameloblastoma, it has been shown that AdAM does not exhibit the KRAS and BRAF mutations seen in these two entities, respectively. Recent report by Bastos et al. [2] identified CTNNB1 hotspot mutations in AdAM, accompanied by nuclear accumulation of beta-catenin. This finding is similar to that seen in dentinogenic ghost cell tumours (DGCT), with which AdAM shares histological, behavioural, and radiological features. CTNNB1 is a key component of the WNT pathway, a major signalling cascade involved in tumorigenesis. This may suggest a histological continuum of WNT-altered benign odontogenic tumours, which includes AdAM and DGCT [6].

It should be noted that it is not always necessary to identify all architectural characteristics indicative of ameloblastoma or AOT. AdAM in contrast to AOT does not harbour the KRAS mutation. Furthermore, in the case of AdAM, the critical finding lies in the presence of local invasion, a feature not typically observed in conventional AOT [7]. Another important distinguishing feature in the current literature is that AdAM typically lacks mutations in the BRAF and p.V600E genes unlike conventional ameloblastoma. Thus, it is important to highlight the difference between these tumours as AdAM is more aggressive when compared to the indolent behaviour of an AOT [1].

In the recent literature, AdAM has been associated with a high recurrence rate of 30% post-surgery [1]. The main reason for this high recurrence is due to the initial misdiagnosis of the tumour as an AOT with subsequent conservative surgical therapy. The high likelihood of misdiagnosis on incisional biopsy is due to predominance of AOT-like features in the areas biopsied. This leads to the use of conservative methods such as enucleation and curettage to treat the lesion [1]. The more definitive approach to management of AdAM is radical resection similar to conventional ameloblastoma as shown in this patient. Only 40% of reported cases have been managed with surgical resection, which further highlights the reason behind the high recurrence rate reported in the literature [1].

In conclusion, while AdAM shares some clinical, radiographic, and histopathologic features with AOT, it is crucial for pathologists and clinicians to thoroughly evaluate all potential features of this tumour. Misidentifying AdAM as AOT can result in under-treatment and higher recurrence rates. Clinicians should recognize that despite its AOT-like appearance, AdAM is more aggressive and should be treated with a radical surgical approach, akin to the treatment of conventional ameloblastoma. The continued documentation and study of AdAM cases will be critical in further refining diagnostic criteria and improving management strategies for this unique entity.
